# Lab to Farm: Applying Research on Plant Genetics and Genomics to Crop Improvement

**DOI:** 10.1371/journal.pbio.1001878

**Published:** 2014-06-10

**Authors:** Pamela C. Ronald

**Affiliations:** 1 Department of Plant Pathology and the Genome Center, University of California, Davis, Davis, California, United States of America; 2 The Joint Bioenergy Institute, Emeryville, California, United States of America; The Sainsbury Laboratory, United Kingdom

## Abstract

Over the last 300 years, plant science research has provided important knowledge and technologies for advancing the sustainability of agriculture. In this Essay, I describe how basic research advances have been translated into crop improvement, explore some lessons learned, and discuss the potential for current and future contribution of plant genetic improvement technologies to continue to enhance food security and agricultural sustainability.

## Abstract 2

In this Essay, Pam Ronald describes how basic research advances have been translated into crop improvement, explores some lessons learned, and discusses the potential for plant genetic improvement technologies to contribute to improved food security and agricultural sustainability.

This article is part of the *PLOS Biology* Collection “The Promise of Plant Translational Research.”

## Introduction

The Earth's human population is expected to increase from the current 6.7 billion to 9 billion by 2050. To feed the growing population, and the 70% increase in the demand for agricultural production that is expected to accompany this increase, a broad range of improvements in the global food supply chain is needed.

There are significant opportunities in plant science research. For example, sustainable agricultural intensification will be important [1] because maintaining current per capita food consumption with no increase in yield, and no decrease in post-harvest and food waste, would necessitate a near doubling of the world's cropland area by 2050 [2],[3]. However, because most of the Earth's arable land is already in production and what remains is being lost to urbanization, salinization, desertification, and environmental degradation, cropland expansion is not a viable approach to food security [4]. Furthermore, because substantial greenhouse gases are emitted from agricultural systems, expansion of cropland would also substantially contribute to carbon mitigation [5]. Thus, the development and deployment of high-yielding crop varieties will make a vital future contribution to sustainable agriculture because it does not rely on expanding cropland.

Water systems are also under severe strain across the world. The fresh water available per person has decreased 4-fold in the last 60 years [4]. Of the water that is available for use, about 70% is already used for agriculture [6]. Many rivers no longer flow all the way to the sea; 50% of the world's wetlands have disappeared and major groundwater aquifers are being mined unsustainably, with water tables in parts of Mexico, India, China, and North Africa declining by as much as 1 meter per year [7]. Thus, increased food production must largely take place on the same land area while using less water. The need for land and water for food production must compete with demands for ecosystem preservation and biomass production.

Compounding the challenges facing agricultural production are the predicted effects of climate change [8]. As the sea level rises and glaciers melt, low lying croplands will be submerged and river systems will experience shorter and more intense seasonal flows, causing more flooding [9]. Yields of our most important food, feed, and fiber crops decline precipitously at temperatures much above 30°C, so heat and drought will also increasingly limit crop production [10]. In addition to these environmental stresses, losses to pests and diseases are also expected to increase. Much of the loss caused by these abiotic and biotic stresses, which already result in 30%–60% yield reductions globally each year, occur after the plants are fully grown; a point at which most or all of the land and water required to grow a crop has been invested [11]. For this reason, a reduction in losses to pests, pathogens, and environmental stresses is equivalent to creating more land and more water [1],[12],[13].

Another important opportunity for increasing food availability is to reduce the amount of food wasted before and after it reaches the consumer (estimated at 30%–50% of total global production) [14]–[16]. Substantial changes in diet through education and/or technological innovation—while difficult—could also make up a good deal of the shortfall in feeding the world's population. For example, a reduction in meat consumption would contribute to increasing the food supply, because 1 hectare of land can produce rice or potatoes for 19–22 people per year whereas the same area will produce enough meat for only 1–2 people.

Augmentation of the nutritional quality of crops is also critical for global food security. Food security, as defined by the Food and Agriculture Organization of the United Nations, “exists when all people, at all times, have physical, social and economic access to sufficient safe and nutritious food that meets their dietary needs and food preferences for an active and healthy life” [17]. Currently, there are 925 million people who are undernourished (∼13% of today's world population), and nearly all live in less developed countries. The long-term effects of malnutrition include stunted growth, learning disabilities, poor health, and chronic disease in later life. Growing more staples that are deficient in essential vitamins and minerals will not tackle health problems caused by nutrient poor diets.

In this Essay, I discuss how discoveries in plant genetic and genomics research can be translated to create new crops and cropping systems that more efficiently use finite resources and that can enhance the quality and quantity of food production. Each strategy must be evaluated in light of its environmental, economic, and social impacts—the three pillars of sustainable agriculture [18].

## What Is Plant Translational Research?

The term plant translational research broadly refers to basic research discoveries that are applied to agronomic improvement. For example, discoveries that reveal basic mechanisms of inheritance in a model plant, such as the genetically tractable plant Arabidopsis, can be applied to crops to accelerate plant breeding [19]. Translational research also encompasses a strategy that has worked well in one crop and then was applied to another. Although not covered in this Essay, translational research also includes non-genetic approaches to improving crop yield or quality emanating from fundamental research on plants, such as research into crop water use efficiency.

## A Brief History of Genetic Improvement

For 10,000 years, we have altered the genetic makeup of our crops, first through primitive domestication and, in the last 300 years, using more sophisticated approaches. For example, in the 1920s, the first hybrid seeds were commercialized. Hybrids inherit their agronomically useful traits, such as high yield, disease resistance, and environmental stress tolerance, from two genetically distinct parents.

Although seeds produced from hybrids can be replanted, they do not have the same combination of beneficial traits as their hybrid parents. For this reason, many farmers who can afford it purchase new hybrid seed each planting season. For farmers who cannot afford hybrid seed or who do not have access to them, it is critical that they have access to improved seed that maintains their parents' advantageous traits when self-pollinated.

Other genetic improvements include mutagenesis—the introduction of random mutations by chemical treatment or radiation, and the interbreeding of related species. Familiar examples of crops generated through interspecific hybridization include many citrus varieties, such as orange varieties, lemon, lime, and grapefruit. The use of wild species as donors of agronomically important traits has also been important to the success of global agriculture [20]. Today virtually everything we eat is produced from seeds that have been genetically altered in one way or another using these well-established approaches of genetic improvement.

## Modern Genetic Technologies

Over the last 20 years, scientists and breeders have used new genetic technologies to develop modern crop varieties. These include marker assisted selection (MAS) and genetic engineering (GE), which have both already led to the development of new crop varieties and which I discuss in more detail below. In addition, new technologies, such as genome editing, have recently emerged as having great promise for crop improvement.

### Marker Assisted Selection

In MAS, researchers first identify the genetic “fingerprint” of the genes that they would like to move from one variety to another, which are usually associated with a desirable trait. Then, two varieties with the desired traits are cross-pollinated, and the breeder identifies those offspring that carry the desirable genetic fingerprint, and eliminates those that don't. The process is then repeated. The advantage of MAS relative to other established plant breeding techniques is that researchers can screen for varieties with the preferred genetic makeup without the need of large field trials, saving both time and labor.

Crops developed through MAS have fewer genetic changes relative to conventionally bred crops because a breeder can track the desired genotype and eliminate undesirable genes at non-targeted loci. For these reasons, the MAS technique is a powerful method for introducing into crop plants traits from their wild relatives and from “primitive” varieties (local domesticates called landraces), which are available from more than 1,700 seed banks worldwide [20].

For example, the development of a new variety of submergence tolerant rice (called Sub1 rice), relied on the existence of an Indian landrace called FR13A (flood resistance 13 A). Although rice can withstand shallow flooding, most rice varieties will die if completely submerged for more than a few days [21]. In Bangladesh and India, four million tons of rice, enough to feed 30 million people, is lost each year to flooding [22]. Using markers found to be linked to the Sub1 locus [23],[24], our team isolated the Sub1 genomic region, which facilitated the development of additional markers [22]. These markers allowed breeders to use MAS to introduce Sub1 into a wide range of rice varieties favored by farmers, while at the same time minimizing the introduction of undesirable traits linked to submergence tolerance in the FR13A donor. The new Sub1 rice varieties are popular in South and Southeast Asia because they are 3-fold higher yielding during periods of flood compared to conventional rice varieties [22].

Currently, many such MAS projects are underway to facilitate the exploration of the genetic variability in our existing food crops to advance crop resilience in the face of the changing climate, pests, and disease.

### Genetic Engineering

“What has long appeared to be simply the agent of a bothersome plant disease is likely to become a major tool for the genetic manipulation of plants: for putting new genes into plants and thereby giving rise to new varieties with desired traits,” wrote acclaimed scientist Mary Dell Chilton in 1983 [25].

Today, more than 30 years later, we can see how the basic research of Chilton, Marc van Montagu, Jeff Schell, and their colleagues, who elucidated the molecular mechanisms with which the bacterial pathogen *Agrobacterium tumefaciens* transfers DNA to plant hosts, has been translated to real-world application—the genetic engineering of plants. In 2012, genetically engineered crops were grown on almost 170 million hectares in 29 countries [26],[27].

To understand why some farmers have embraced GE crops and how they benefit the environment [28], consider Bt cotton, which contains a bacterial protein called Bt that kills pests, such as the cotton bollworm, without harming beneficial insects and spiders. Bt is benign to humans, which is why organic farmers have used Bt sprays and other formulations as their primary method of pest control for 50 years [29],[30]. Although Bt insecticides are permitted in organic farming, Bt crops are not, because the National Organic Program standards in the US and other countries prohibit the use of GE crops in organic agriculture.

In 2012, 70%–90% of American, Indian, and Chinese farmers grew Bt cotton [31]. A team of Chinese and French scientists reported that widespread planting of Bt cotton in China drastically reduced the use of synthetic insecticides [32], increased the abundance of beneficial organisms on farms, and decreased populations of crop-damaging insects [30],[32],[33]. Its cultivation in China has also reduced pesticide poisoning in farmers and their families [33]. US farms that have cultivated Bt cotton have twice the insect biodiversity relative to neighboring conventional farms. In India, farmers growing Bt cotton increased their yields by 24%, their profits by 50%, and raised their living standards by 18%, according to one common standard that measures household expenditures [34]. The economic benefits of planting Bt cotton extend beyond the farm and into the community; for example, M. Qaim and colleagues reported that villages in India that planted Bt cotton received net increases in income at all social levels, not only farmers, and that women have particularly benefited from its adoption [35]–[40]. Similarly, insecticide use on US corn farms declined 10-fold from 1995 to 2010, consistent with the steady decline in European corn borer that is a direct result of Bt adoption [83].

GE papaya [41], engineered to withstand a devastating viral infection, has also been rapidly adopted and is now grown by nearly all Chinese and Hawaiian papaya farmers [26],[31],[41]. It carries a snippet of viral genome, which immunizes it against infection. There is currently no other method—organic or conventional—to adequately control this disease. Yields of GE papaya have increased 10 to 20-fold relative to conventional and organic papaya.

### Genome Editing

A more recent technology, called genome editing, which makes it possible to precisely alter DNA sequences in living cells, is expected to lead to new crop varieties in the near future [42]. In this technique, targeted double-strand DNA breaks are introduced in the genome at or near the site where a DNA sequence modification is desired using sequence-specific nucleases. The repair of the break can be used to introduce specific DNA sequence changes, DNA deletions, or even serve as an insertion site for arrays of transgenes.

Genome editing can thus be used to introduce genetic variation without transgenesis, and can even be used to recreate naturally occurring mutations into elite varieties of crops. For this reason, some scientists and farmers believe that crops generated through this technology will prove to be more socially acceptable in Europe and elsewhere than those generated by genetic engineering.

As discussed in the accompanying essay [42], genome editing has been used to engineer rice for resistance to the bacterial pathogen, *Xanthomonas oryzae* pv. *oryzae*. Researchers created mutations in the promoter of a rice sucrose-efflux transporter gene, which is targeted by a pathogen effector [43],[44]. These mutations, which are mostly DNA deletions, eliminated the transcriptional induction required for pathogen virulence, rendering the plant resistant [45].

### Other Approaches

Another technique for introducing genetic variation is induced mutagenesis through chemical or radiation treatment [46]. A recent variation on mutagenesis, called Targeted Induced Local Lesions in Genomes (TILLING), facilitates the identification and deployment of gene variants that encode agronomically important traits. This approach has been particularly useful for improving understudied crops. For example, melon variants have been identified through TILLING that have improved shelf life [47] and those with unisexual flowers [48], traits that can enhance productivity in India. Another example is the identification of acyanogenic sorghum variants that can be used as improved animal fodder [49].

Genetically improved seed, whether derived from conventional genetic modification or newly developed technologies such as genome editing, must be integrated into ecologically based farming systems (see Box 1) to maximize their impact on enhancing sustainable agriculture and food security [50]–[56].

**Box 1. A Complementary and Vital Role for Agroecological Farming Practices**

The cultivation of genetically improved crops must be integrated into ecologically based farming systems to maximize their impact on enhancing sustainable agriculture and food security. Farmers cannot rely on seed alone to eliminate pests. For example, deployment of a “refuge strategy”—creating refugia of crop plants that do not make Bt toxins—promotes the survival of susceptible insects and helps to delay the evolution of pest resistance to Bt crops [50]. Whereas this approach has been successful in the US, where farmers are required to plant refugia, failure to provide adequate refugia appears to have hastened pink bollworm resistance in India. Similarly, where Bt maize has been planted continuously without rotation with other crops, western corn rootworm has evolved resistance to Bt [51]. These examples emphasize the need to deploy crop rotation and diversity to reduce the evolution of insect resistance.

Farmers face similarly complex issues when controlling weeds. Cotton, corn, soybean, and sugar beet crops have been genetically engineered for resistance to a herbicide called glyphosate [52]. The adoption of such herbicide tolerant (HT) crops has enabled farmers to substitute glyphosate for more toxic and persistent herbicides [83], reduced the need for ploughing, reduced soil erosion [53], water loss [54], and greenhouse gas emissions [5], and enhanced soil health (more carbon and nutrients kept in soil). In 2005, the decreased tillage that accompanied planting of HT soybeans was equivalent to removing 4 million cars from the roads [55].

A drawback to the popularity of this approach (80%–90% of the cotton, corn, soybeans, and sugar beets grown by US farmers is an HT crop) is that it has led to reliance on a single herbicide resulting in the evolution of 24 glyphosate-resistant weed species since HT crops were introduced in 1996 [56]. HT crops developed through conventional breeding have suffered the same fate [56], as will crops developed through genome editing unless farmers couple HT seed with integrated strategies to manage weeds.

## Translating Basic Research to Benefit Subsistence Farmers

Despite the considerable and continuing breakthroughs in plant genetic and genomic technologies, there has been relatively little global government investment into funding basic plant science and in translating these discoveries into food crops beneficial to farmers in less developed countries.

To fill the gap, some foundations and public–private partnerships have launched programs. For example, the Bill and Melinda Gates Foundation is supporting a large program, called *Stress-Tolerant Rice for Africa and South Asia* [57], which is assisting with the development and dissemination of the Sub1 rice variety, which resulted from a ten-year basic research collaboration funded primarily by the US Department of Agriculture. With the help of the Gates Foundation, last year more than 4 million farmers grew Sub1 rice [22].

The Rockefeller Foundation was instrumental in funding the development of Golden Rice [58], a genetically engineered rice enriched for provitamin A that is expected to be released soon [59]. Worldwide, over 124 million children are vitamin A-deficient; many go blind or become ill from diarrhea, and nearly 8 million preschool-age children die each year as the result of this deficiency. A public–private partnership advanced the development of second generation Golden Rice [60],[61]. One report estimates that improved vitamin A nutritional status obtained from eating vitamin A rice could prevent the deaths of thousands of young children each year [62]. The positive effects of Golden Rice are predicted to be most pronounced in the lowest income groups at a fraction of the cost of the current supplementation programs [62],[63], which are not only costly to run but also not always continued [58].

The Water Efficient Maize for Africa (WEMA) project is another important public–private partnership, which aims to develop drought-tolerant and insect-protected maize using conventional breeding, MAS, and biotechnology. The goal is to make these varieties available royalty free to small-hold farmers in sub-Saharan Africa through African seed companies [64]. The introduction of drought-tolerant maize to Africa, where three-quarters of the world's severe droughts have occurred over the past ten years, is predicted to dramatically increase yields of this staple food crop for local farmers [64],[65].

Another exciting development is the US Agency for International Development (USAID) “Feed the Future” program, which partners with diverse countries to enhance local food security [66]. For example the Maharashtra Hybrid Seed Company and Cornell University have jointly developed Bt eggplant that is resistant to fruit and shoot borers [27]. Bt eggplant was recently made available on a royalty-free basis to smallholder farmers in Bangladesh. Researchers estimate that farmers growing the new Bt eggplant varieties could obtain yield increases of 30%–45% while reducing insecticide use.

The USAID has also funded projects to enhance the productivity of banana, a staple food crop for more than 100 million people in East Africa, and which is susceptible to several serious diseases. Many strategies to control this disease rely on genetic engineering because most bananas don't produce seed and are propagated clonally [67]–[70]. Bananas with resistance to banana *Xanthomonas* wilt disease (BXW), have recently been genetically engineered with the rice XA21 resistance gene [71].

These examples demonstrate the success of non-profit and public–private partnerships in translating basic research discoveries into benefits at the farm. Well-funded, long-term, multinational, multidisciplinary collaborations are vital if we are to continue making significant progress in developing new crop varieties to enhance food security in the developing world. In a recent report, leading scientists highlighted the need for significant investment in plant breeding and estimated that US$200 million annually is needed to carry out such a systematic, concerted, collaborative global effort [20].

## Conclusions

Despite the scientific consensus that the genetically engineered crops on the market are safe to eat, have massively reduced the use of sprayed insecticides, and have benefited the environment, they are still viewed with skepticism by some consumers [49]. Without public support for genetic technologies, regulatory costs will continue to climb. The end result may be that only multinational corporations can afford to develop and license such crops [72]. This exclusivity places constraints on broad access to genetic technologies because large corporations have little incentive to develop subsistence (e.g., cassava and banana) and specialty crops (e.g., strawberries, apples, lettuce)—for poor farmers that need them. Costly regulations also hinder the creation of small businesses that wish to translate discoveries in plant genetics into commercially viable enterprises.

To reduce regulatory costs, many scientists and regulators in the US and Europe advocate for a trait-based, regulatory approval process that would assess the benefits associated with a new crop variety, as well as the risks and costs of not adopting a particular variety. The advantage of this approach is that it would advance the deployment of agricultural technologies that could contribute to sustainable agriculture. Currently, GE crops are regulated on the basis of the technology used to generate them.

A related issue, which applies to most seed developed by corporations (conventional or genetically engineered) [73]), is that intellectual property rights constrain sharing of genetic resources. Whereas seeds protected by the plant variety protection act include exemptions for farmers to save seed for next year's planting and for breeders to include the variety in breeding programs, certain plant varieties, including GE crops, can be protected by patents, which are much more restrictive and prohibit seed saving by farmers and breeders [73].

The US Supreme Court recently affirmed that farmers are not permitted to reproduce patented seeds through planting and harvesting without the patent owner's permission [74]. Whether the principle of patenting genes is morally or ethically correct is a matter of intense debate [75]. There are those who see all biological material as a public good or a gift from nature and, therefore, something that cannot be owned by an individual or company. Some fear that patenting will restrict inventions and progress in breeding if germplasm and genes are removed from the public domain. Others see patents as a spur to the process of discovery and development of socially beneficial products.

Although ∼25% of the patented inventions in agricultural biotechnology were made by public sector researchers (e.g., public universities), many of these inventions are exclusively licensed to private companies [76]. Five firms (Monsanto, Dupont-Pioneer, Syngenta, Bayer, BASF) produce the majority of the world's seeds and control many of the older technologies such as Bt and transformation [77]. Fortunately, the business landscape is changing as many of the earlier patents expire or as alternatives to enabling technologies controlled by corporations emerge in the public sector and as more countries use genetic engineering to create a greater variety of crops. The European Commission predicts that in the near future, half of the new GE crops will come from national technology providers in Asia and Latin America that are designed for domestic markets [78]. The reduced dominance of multi-national seed companies may alleviate concerns of consumers, some of whom oppose modern plant genetics because they see it as a tool of large corporations.

University scientists have also been active in reversing the trend of exclusively licensing genetic technologies to a few corporations that control most of the world's seed production [79],[80]. For example, the Rockefeller and McKnight Foundations joined leading US agricultural universities and plant research institutes to establish the Public Intellectual Property Resource for Agriculture (PIPRA). PIPRA helps universities to retain rights of their technologies for humanitarian purposes and for crops that are vital to small-acreage farmers. The goal is to create an agricultural and food system that is directed broadly at the public good, not one dominated by private interests.

Ultimately, the continued translation of basic research into tangible crop improvement will rely not only on the research itself but also in communicating the vital role that agriculture and plant genetics plays in all of our lives. In the developed world where less than 2% of the population are farmers, the challenges of producing food in a sustainable manner is far removed from the average consumer. In our role as educators, plant biologists can promote agricultural literacy through the establishment of elementary and university curriculums that highlight the social, economic, biological, environmental, and ethical aspects of food production. We can more fully engage with the policy makers, non-governmental organizations, and journalists by providing science-based information in more creative ways—for example through social media and videography. Many such efforts are now being launched around the globe [81],[82]. An engaged, informed public will help us to attain an agricultural system that can produce safe food in a secure, sustainable, and equitable manner.

## Abbreviations

MAS: marker assisted selection
GE: genetic engineering

## References

[1] Royal Society T (2009) Reaping the benefits: science and the sustainable intensification of global agriculture. Society TR, editor. London: The Royal Society.

[2] Green RE, Cornell SJ, Scharlemann JPW, Balmford A (2005) Farming and the fate of wild nature. Science 307: 550–555. 15618485 10.1126/science.1106049

[3] Waggoner PE (1995) How much land can ten billion people spare for nature? Does technology make a difference? Technol Soc 17: 17–34.

[4] UNEP Global Environment Outlook 3 (2002) Global Environment Outlook 3. New York: United Nations.

[5] Burney JA, Davis SJ, Lobell DB (2010) Greenhouse gas mitigation by agricultural intensification. Proc Natl Acad Sci U S A 107: 12052–12057. 20551223 10.1073/pnas.0914216107 PMC2900707

[6] Vorosmarty CJ, Green P, Salisbury J, Lammers RB (2000) Global water resources: vulnerability from climate change and population growth. Science 289: 284–288. 10894773 10.1126/science.289.5477.284

[7] Somerville C, Briscoe J (2001) Genetic engineering and water. Science 292: 2217. 11423621 10.1126/science.292.5525.2217

[8] Lobell DB, Burke MB, Tebaldi C, Mastrandrea MD, Falcon WP, et al (2008) Prioritizing climate change adaptation needs for food security in 2030. Science 319: 607–610. 18239122 10.1126/science.1152339

[9] Intergovernmental Panel on Climate Change (2007) Climate change 2007: impacts, adaptation and vulnerability. Parry ML, Canziani OF, Palutikof JP, van der Linden PJ, Hanson CE, editors. Cambridge: Cambridge University Press. 976 p.

[10] Schlenker W, Roberts MJ (2009) Nonlinear temperature effects indicate severe damages to U.S. crop yields under climate change. Proc Natl Acad Sci U S A 106: 15594–15598. 19717432 10.1073/pnas.0906865106 PMC2747166

[11] Dhlamini Z, Spillane C, Moss JP, Ruane J, Urquia N, et al. (2005) Status of research and application of crop biotechnologies in developing countries. Rome: Food and Agriculture Oganization of the United Nations Natural Resources Management and Environment Department

[12] Gregory PJ, Johnson SN, Newton AC, Ingram JSI (2009) Integrating pests and pathogens into the climate change/food security debate. J Exp Bot 60: 2827–2838. 19380424 10.1093/jxb/erp080

[13] World Bank (2007) Agriculture for development. World Bank, editor. World Development Report. Washington (D.C.): The World Bank.

[14] Institution of Mechanical Engineers (IMechE) (2013) Feeding the 9 billion: The tragedy of waste. Available: http://www.imeche.org/knowledge/themes/environment/global-food. Global Food Waste Not, Want Not.

[15] Gustavsson J, Cederberg C, Sonesson U, Otterdijk Rv, Meybeck A (2011) Global food losses and food waste: extent, causes and prevention. Rome: FAO.

[16] Lundqvist J, de Fraiture C, Molden D (2008) Saving water: from field to fork - curbing losses and wastage in the food chain. Stockholm: Stockholm International Water Institute (SIWI) Policy Brief.

[17] FAO (2014) Committee on World Food Security (CFS) Available: http://www.fao.org/cfs/cfs-home/en/

[18] Committee on the Impact of Biotechnology on Farm-Level Economics and Sustainability and The National Research Council T (2010) Impact of genetically engineered crops on farm sustainability in the United States. Washington (D.C.): The National Academies Press.

[19] Comai L (2014) Genome elimination: translating basic research into a future tool for plant breeding. PLoS Biol 12: e1001876. 24915001 10.1371/journal.pbio.1001876 PMC4051579

[20] McCouch S, Baute GJ, Bradeen J, Bramel P, Bretting PK, et al (2013) Feeding the future. Nature 499: 23–24. 23823779 10.1038/499023a

[21] Xu K, Xu X, Fukao T, Canlas P, Maghirang-Rodriguez R, et al (2006) Sub1A is an ethylene response-factor-like gene that confers submergence tolerance to rice. Nature 442: 705–708. 16900200 10.1038/nature04920

[22] Xu K, Ismail A, Ronald P (2013) Flood tolerance mediated by the rice Sub1A transcription factor 1. Jenks M, Hasegawa P, editors. Plant abiotic stress, 2nd edition. Ames (Iowa): Wiley-Blackwell.

[23] Xu K, Xu X, Ronald P, Mackill DJ (2000) A high-resolution linkage map in the vicinity of the rice submergence tolerance locus Sub1. Mol Genet Genomics 263: 681–689. 10.1007/s004380051217 10852491

[24] Xu KN, Mackill DJ (1996) A major locus for submergence tolerance mapped on rice chromosome 9. Mol Breeding 2: 219–224.

[25] Chilton MD (1983) A vector for introducing new genes into plants. Sci Am 248: 36–45.6823543

[26] James C (2012) Global status of commercialized biotech/GM crops: 2012. Ithaca (New York): ISAAA.

[27] USAID (2004) Pest resistant eggplant: India, Bengladesh and Phillipines Available: http://pdf.usaid.gov/pdf_docs/pdacp867.pdf). Washington (D.C.): USAID.

[28] Carpenter JE (2010) Peer-reviewed surveys indicate positive impact of commercialized GM crops. Nat Biotechnol 28: 319–321. 20379171 10.1038/nbt0410-319

[29] Roh JY, Choi JY, Li MS, Jin BR, Je YH (2007) Bacillus thuringiensis as a specific, safe, and effective tool for insect pest control. J Microbiol Biotechn 17: 547–559. 18051264

[30] Tabashnik BE, Brevault T, Carriere Y (2013) Insect resistance to Bt crops: lessons from the first billion acres. Nat Biotechnol 31: 510–521. 23752438 10.1038/nbt.2597

[31] James C (2013) Global status of commercialized biotech/GM crops: 2013. Ithaca (New York): ISAAA.

[32] Lu Y, Wu K, Jiang Y, Guo Y, Desneux N (2012) Widespread adoption of Bt cotton and insecticide decrease promotes biocontrol services. Nature 487: 362–365. 22722864 10.1038/nature11153

[33] Pray CE, Huang JK, Hu RF, Rozelle S (2002) Five years of Bt cotton in China - the benefits continue. Plant Journal 31: 423–430. 12182701 10.1046/j.1365-313x.2002.01401.x

[34] Kathage J, Qaim M (2012) Economic impacts and impact dynamics of Bt (Bacillus thuringiensis) cotton in India. Proc Natl Acad Sci U S A 109: 11652–11656. 22753493 10.1073/pnas.1203647109 PMC3406847

[35] Qaim M, Subramanian A (2010) Benefits of transgenic plants: a socioeconomic perspective. Kempken F, Lorz H, Nagata T, editors. Genetic modification of plants: agriculture, horticulture and forestry. pp. 615–629.

[36] Qaim M, Subramanian A, Sadashivappa P (2009) Commercialized GM crops and yield. Nat Biotechnol 27: 803–804. 19741630 10.1038/nbt0909-803b

[37] Subramanian A, Kirwan K, Pink D (2010) Food security: perception failures. Science 328: 173–173. 20378801 10.1126/science.328.5975.173-b

[38] Qaim M, Subramanian A, Sadashivappa P (2010) Socioeconomic impacts of Bt (*Bacillus thuringiensis*) cotton. Biotechnology in agriculture and forestry. New York: SpringerLink.

[39] Subramanian A, Qaim M (2010) The impact of Bt cotton on poor households in rural India. J Dev Stud 46: 295–311.

[40] Subramanian A, Kirwan K, Pink D, Qaim M (2010) GM crops and gender issues. Nat Biotechnol 28: 404–406. 20458298 10.1038/nbt0510-404

[41] Tripathi S, Suzuki J, Gonsalves D (2007) Development of genetically engineered resistant papaya for papaya ringspot virus in a timely manner: a comprehensive and successful approach. Methods Mol Biol 354: 197–240. 17172756 10.1385/1-59259-966-4:197

[42] Voytas D, Gao C (2014) Precision genome engineering and agriculture: opportunities and regulatory challenges. PLoS Biol 12: e1001877. 24915127 10.1371/journal.pbio.1001877 PMC4051594

[43] Chen LQ, Hou BH, Lalonde S, Takanaga H, Hartung ML, et al (2010) Sugar transporters for intercellular exchange and nutrition of pathogens. Nature 468: 527–532. 21107422 10.1038/nature09606 PMC3000469

[44] Yang B, Sugio A, White FF (2006) Os8N3 is a host disease-susceptibility gene for bacterial blight of rice. Proc Natl Acad Sci U S A 103: 10503–10508. 16798873 10.1073/pnas.0604088103 PMC1502487

[45] Li T, Liu B, Spalding MH, Weeks DP, Yang B (2012) High-efficiency TALEN-based gene editing produces disease-resistant rice. Nat Biotechnol 30: 390–392. 22565958 10.1038/nbt.2199

[46] Ahloowalia BS, Maluszynski M, Nichterlein K (2004) Global impact of mutation-derived varieties. Euphytica 135: 187–204.

[47] Dahmani-Mardas F, Troadec C, Boualem A, Leveque S, Alsadon AA, et al (2010) Engineering melon plants with improved fruit shelf life using the TILLING approach. PLoS ONE 5: e15776. 21209891 10.1371/journal.pone.0015776 PMC3012703

[48] Foucart C, Boualem A, Lasseur B, Eleblu J, Fahraj I, et al (2012) Sex determination in cucurbits. Biologie Aujourd hui 206: 57–62. 22463996 10.1051/jbio/2012005

[49] Blomstedt CK, Gleadow RM, O'Donnell N, Naur P, Jensen K, et al (2012) A combined biochemical screen and TILLING approach identifies mutations in Sorghum bicolor L. Moench resulting in acyanogenic forage production. Plant Biotechnol J 10: 54–66. 21880107 10.1111/j.1467-7652.2011.00646.x

[50] Tabashnik B, Gassman AJ, Crowder DW, Carriere Y (2008) Insect resistance to *Bt* crops: evidence versus theory. Nat Biotechnol 26: 199–202. 18259177 10.1038/nbt1382

[51] Gassmann AJ, Petzold-Maxwell JL, Clifton EH, Dunbar MW, Hoffmann AM, et al (2014) Field-evolved resistance by western corn rootworm to multiple Bacillus thuringiensis toxins in transgenic maize. Proc Natl Acad Sci U S A 111: 5141–5146. 24639498 10.1073/pnas.1317179111 PMC3986160

[52] Kegley SE, Hill BR, Orme S, Choi AH (2010) PAN Pesticide Database, Pesticide Action Network, North America. San Francisco. Available: http:wwwpesticideinfoorg.

[53] Givens WA, Shaw DR, Kruger GR, Johnson WG, Weller SC, et al (2009) Survey of tillage trends following the adoption of glyphosate-resistant crops. Weed Technol 23: 150–155.

[54] US Soybean Export Council (2013) US Soy - a sustainability system that delivers. US Soybean farmers sustainability report. Available: http://28vp741fflb42av02837961yayh.wpengine.netdna-cdn.com/wp-content/uploads/2012/09/13-USSEC-0147_Annual-Report-10-23-13.pdf).

[55] Brookes G, Barfoot P (2006) Global impact of biotech crops: socio-economic and environmental effects in the first ten years of commercial use. AgBio Forum 9: 139–151.

[56] Gilbert N (2013) Case studies: a hard look at GM crops. Nature (News Feature) 497: 24–26. 10.1038/497024a 23636378

[57] IRRI (2007) Stress-tolerant rice for Africa and South Asia. Available: https://sites.google.com/a/irri.org/strasa/. International Rice Research Institute and AfricaRice.

[58] Golden Rice Humanitarian Board (2005) Golden Rice Project. Available: http://www.goldenrice.org/index.php. Rockefeller Foundation.

[59] Harmon A (2013 August 24) Golden rice: lifesaver? The New York Times Sunday Review

[60] Paine JA, Shipton CA, Chaggar S, Howells RM, Kennedy MJ, et al (2005) Improving the nutritional value of Golden Rice through increased pro-vitamin A content. Nat Biotechnol 23: 482–487. 15793573 10.1038/nbt1082

[61] Tang G, Qin J, Dolnikowski GG, Russell RM, Grusak MA (2009) Golden Rice is an effective source of Vitamin A. Am J Clin Nutr 89: 1776–1783. 19369372 10.3945/ajcn.2008.27119 PMC2682994

[62] Stein AJ, Sachdev HPS, Qaim M (2006) Potential impact and cost-effectiveness of Golden Rice. Nat Biotechnol 24: 1200–1201. 17033649 10.1038/nbt1006-1200b

[63] Stein AJ, Sachdev HPS, Qaim M (2008) Genetic engineering for the poor: Golden Rice and public health in India. World Dev 36: 144–158.

[64] African Agricultural Technology Foundation (2012) Water efficient maize for Africa (WEMA). Available: http://wema.aatf-africa.org/aatf-projects/water-efficient-maize-africa-wema). African Agricultural Technology Foundation (AATF-Africa).

[65] African Agricultural Technology Foundation T (2010) Scientists prepare for confined field trials of life-saving drought-tolerant transgenic maize. Nairobi: African Agricultural Technology Foundation (AATF).

[66] USAID (2012) Feed the future: the U.S. government's global hunger and food security initiative. Available: http://feedthefuture.gov/about. Washington (D.C.): USAID.

[67] Peed M (2011 January 10) We have no bananas: can scientists defeat a devastating blight? The New Yorker 21717800

[68] Studholme DJ, Kemen E, MacLean D, Schornack S, Aritua V, et al (2010) Genome-wide sequencing data reveals virulence factors implicated in banana *Xanthomonas* wilt. FEMS Microbiol Lett 310: 182–192. 20695894 10.1111/j.1574-6968.2010.02065.x

[69] Tripathi L, Mwangi M, Abele S, Aritua V, Tushemereirwe WK, et al (2009) *Xanthomonas* Wilt: a threat to banana production in East and Central Africa. Plant Disease 93: 440–451. 10.1094/PDIS-93-5-0440 30764143

[70] FAO (2004) Banana improvement. FAO Corporate Document Repository, editor. Available: http://www.fao.org/docrep/007/ae216e/ae216e01.htm. Agriculture and Consumer Protection.

[71] Tripathi JN, Lorenzen J, Bahar O, Ronald PC, Tripathi L (2014) Transgenic expression of the rice *Xa21* pattern recognition receptor in banana (Musa sp.) confers resistance to *Xanthomonas campestris* pv. *musacearum* . Plant Biotechnol J In press. 10.1111/pbi.12170 PMC4110157 24612254

[72] Miller JK, Bradford KJ (2010) The regulatory bottleneck for biotech specialty crops. Nat Biotechnol 28: 1012–1014. 20944582 10.1038/nbt1010-1012

[73] Leyser O (2014) Moving beyond the GM debate. PLoS Biol 12: e1001887 doi:10.1371/journal.pbio.1001887 24914954 10.1371/journal.pbio.1001887 PMC4051613

[74] United States Supreme Court (2013) Bowman v. Monsanto Co. et al. Certiorari to the United States Court of Appeals for the Federal Circuit No. 11-796 (Argued February 19, 2013–Decided May 13, 2013).

[75] Gladwell M (1995 November 13) Rights to life: are scientists wrong to patent genes? New Yorker 11654950

[76] Atkinson RC, Beachy RN, Conway G, Cordova FA, Fox MA, et al (2003) Public sector collaboration for agricultural IP management. Science 301: 174–175. 12855794 10.1126/science.1085553

[77] Toenniessen G (2006) Opportunities for and challenges to plant biotechnology adoption in developing countries. Available: http://www.rockfound.org/Library/Opportunities_for_and_Challenges_to_Plant_Biotechnology_Adoption.pdf. National Agricultural Biotechnology Council meeting, Science and Society at a Crossroad.

[78] European Commission Joint Research Centre (2008) Scientific and technical contribution to the development of an overall health strategy in the area of GMOs. European Commission Joint Research Center, editor. Ispra, Italy: European Commission

[79] Ronald PC (1998) Genetic Resources Recognition Fund. AgBiotech News and Information 10: 19N–21N. CAB International

[80] ten Kate K, Collis A (1997) The genetic resources recognition fund of the University of California, Davis. Executive Secretary of the Convention on Biological Diversity by the Royal Botanic Gardens, Kew Benefit Sharing Case Study.

[81] Public Schools of North Carolina (2012) Race to the top contract alert - NCSSM Public Notice 2011–2012. Available: http://www.ncpublicschools.org/publicnotices/notices/2011-12/20120606-01. Department of Public Instruction, editor. North Carolina: State Board of Education.

[82] Public Schools of New York (2012) Race to the top contract alert. Department of Public Instruction, editor. New York: State Board of Education.

[83] Fernandez-Cornejo J, Wechsler S, Livingston M, Mitchell L (2014) Genetically engineered crops in the United States. USDA Economic Research Report No. (ERR-162) 60 pp, February.

